# Reducing the immediate availability of red blood cells in cardiac surgery, a single-centre experience

**DOI:** 10.1007/s12471-014-0618-9

**Published:** 2014-10-18

**Authors:** M. C. Haanschoten, A. H. M. van Straten, F. Verstappen, D. van de Kerkhof, A. A. J. van Zundert, M. A. Soliman Hamad

**Affiliations:** 1 Department of Anesthesiology, Catharina Hospital, Eindhoven, the Netherlands; 2 Intensive Care Unit, Catharina Hospital, Eindhoven, the Netherlands; 3 Department of Cardio-Thoracic Surgery, Catharina Hospital, Michelangelolaan 2, Postbus 1350, 5602 ZA Eindhoven, the Netherlands; 4 Clinical laboratory, Catharina Hospital, Eindhoven, the Netherlands; 5 Department of Anesthesiology, University Hospital Ghent, Ghent, Belgium; 6 University Hospital Maastricht, Maastricht, the Netherlands

**Keywords:** Cardiac surgery, Red blood cells, Transfusion

## Abstract

**Background:**

In our institution, we have redefined our criteria for direct availability of red blood cell (RBC) units in the operation room. In this study, we sought to evaluate the safety of applying this new logistical policy of blood transfusion in the first preliminary group of patients.

**Methods:**

In March 2010, we started a new policy concerning the elective availability of RBC units in the operation room. This policy was called: No Elective Red Cells (NERC) program. The program was applied for patients undergoing primary isolated coronary artery bypass grafting (CABG) or single valve surgery. No elective RBC units were preoperatively ordered for these patients. In case of urgent need, blood was delivered to the operating room within 20 min. The present study includes the first 500 patients who were managed according to this policy. Logistic regression analyses were performed to investigate the impact of biomedical variables on fulfilling this NERC program.

**Results:**

The majority of patients (*n* = 409, 81 %) did not receive any RBCs during the hospital stay. In patients who did receive RBCs (*n* = 91, 19 %), 11 patients (2.2 %) received RBCs after 24 h postoperatively. Female gender, left ventricular ejection fraction (LVEF) and EuroSCORE were significant predictors for the need of blood transfusion (OR = 3.12; 2.79; 1.17 respectively).

**Conclusion:**

In a selected group of patients, it is safe to perform cardiac surgery without the immediate availability of RBCs in the operating room. Transfusion was avoided in 81 % of these patients. Female gender, LVEF and EuroSCORE were associated with blood transfusion.

## Introduction

Perioperative transfusion of red blood cells (RBCs) has been associated with increased mortality, morbidity, costs and decreased long-term survival after cardiac surgery [1, 2]. The severity of the adverse effects of perioperative RBC transfusion is dose-dependent. Unnecessary transfusion might cause postoperative complications and subsequent hospitalisation costs [1, 3]. In an earlier report [4], we identified the independent, patient-related risk factors of RBC transfusion after coronary artery bypass grafting (CABG). Identification of these risk factors led us to follow a new policy concerning the direct availability of packed RBCs in the operating theatre. It was standard practice in our hospital to have two units of RBCs directly available in the operating theatre for every patient who is scheduled for an elective CABG. If these two units are not used, they are returned to the laboratory to be used for another patient. The quality of these RBCs is sometimes reduced and the RBCs cannot be used anymore. Since March 2010, we have followed a policy that is called: No Elective Red Cells (NERC) protocol. With this new protocol, in a selected group of patients, no units of RBCs are directly available in the operating theatre. According to this protocol, RBCs that are available in the blood bank are delivered into the operating room within 20 min after request.

In the present study, we have analysed our results of the first consecutive 500 patients who were managed according to the NERC protocol.

## Materials and methods

### The NERC protocol

In our institution, we have started a new strategy concerning direct availability of RBC units in the operating room for a selected group of patients undergoing cardiac surgery. In this group of patients, only ABO typing and screening for irregular antibodies without cross matching were performed. Units of RBCs were kept in the hospital’s blood bank. All patients were examined in the preoperative screening in the outpatient clinic to select suitable candidates for the NERC program. For patients included in the protocol, no RBCs were ordered before the operation. Patients included in the NERC program were screened for blood type and irregular antibodies the day before surgery. In case of urgent need for transfusion of RBCs during or immediately after the operation, the local blood bank prepared the RBCs, including electronic cross matching, and delivered them within 20 min. Uncross-matched blood was not given to any of the patients in this population.

We always perform a double ABO screening including the Rh typing. In addition, we performed one screening on irregular antibodies for every patient. These tests are valid for 48 h. Afterwards, a double ABO and antibody screenings should be repeated.

### Patients

The study included the first consecutive 500 patients who were managed according to the NERC protocol starting in March 2010. Approval was obtained from the institution’s research review board that waived the need for informed consent. Data of demographics, operative techniques, blood transfusions and adverse events were prospectively collected in the computerised database of our department.

Patients were managed according to the NERC protocol when they fulfilled the following criteria:

- Isolated primary coronary artery bypass grafting (CABG), isolated aortic valve replacement (AVR) or isolated mitral valve surgery.
- Preoperative haemoglobin (Hb) level of >7.0 mmol/L (11.3 g/dL).
- Body surface area (BSA) of >1.7 m^2^.
- Negative test for irregular antibodies.

Patients were excluded from the NERC protocol in case of the following criteria:

- Combined CABG and/or valve surgery.
- Prior cardiac surgery.
- Positive test for irregular antibodies.

### Operative technique

All patients received short-acting anaesthetic drugs to facilitate early extubation and rapid recovery according to our daily practice. Normothermic extracorporeal circulation (ECC) was performed using non-pulsatile flow. Cold crystalloid cardioplegia (St Thomas’ solution) or warm blood cardioplegia was used to induce and maintain cardioplegic cardiac arrest, according to the surgeon’s preference. Cell saver was used to collect all shed and/or residual blood, which was retransfused immediately after the operation.

### Indications of RBC transfusion

In our hospital, transfusion of one or more units of RBC is indicated in the postoperative period in case of a haemoglobin level of <5 mmol/L (8 g/dL) or a haematocrit value of <0.25. A haemoglobin level of <6 mmol/L (9.5 g/dL) is adopted in case of haemodynamically unstable patients or patients with postoperative ischaemia or excessive bleeding. If the patient did not fulfil any of these criteria, no blood transfusion was given.

### Statistics

Continuous variables are expressed as mean ± SD and categorical data are expressed as numbers (percentage). Univariate logistic regression analyses were performed to investigate the impact of biomedical variables on fulfilling the NERC protocol. A *P*-value < 0.05 was used for all tests to indicate statistical significance. Odds ratios (OR) with a confidence interval (CI) of 95 % with p-values are reported. All statistical analyses were performed using SPPS version 17.0 (SPSS Inc, Chicago, IL).

## Results

Starting in March 2010, 500 consecutive patients underwent cardiac surgery in our hospital and fulfilled the criteria for the NERC protocol. Demographic data and comorbidities are shown in Table 1. The majority of patients were male (82 %). The following comorbidities were present: diabetes (19 %), hypertension (52.8 %), severe renal function impairment (1.2 %), chronic obstructive pulmonary disease (COPD) (9 %), peripheral vascular disease (PVD) (13.6 %), history of preoperative atrial fibrillation (4.4 %) and preoperative myocardial infarction (37.6 %). The mean logistic EuroSCORE was 3.2 ± 3.3 and the mean additive EuroSCORE was 3.1 ± 2.3.

**Table 1:** Demographic data and comorbidities

Variable	Incidence
Male gender	411 (82.2 %)
Age	63.8 ± 9.7 (29–87)
Diabetes	93 (19 %)
Hypertension	264 (52.8 %)
Serum haemoglobin (mmol/L)	8.7 ± 0.7 (7.0–10.7)
Serum creatinine (mmol/L)	94.6 ± 27.8 (54–390)
Serum creatinine >200 mmol/L	6 (1.2 %)
COPD	45 (9 %)
PVD	68 (13.6 %)
Atrial fibrillation	22 (4.4 %)
Previous MI	182 (37.6 %)
LVEF <35 %	19 (3.8 %)
Logistic EuroSCORE	3.2 ± 3.3 (1–35)
Additive EuroSCORE	3.1 ± 2.3 (0–11)

Data are presented as numbers (%) or mean ± SD

*COPD* chronic obstructive pulmonary disease, *LVEF* left ventricular ejection fraction, *MI* myocardial infarction, *PVD* peripheral vascular disease

Table 2 shows the type of the operations performed. The majority of patients underwent coronary artery bypass grafting (CABG) including on pump (74 %) and off pump (OPCAB) (12 %) surgery. An overview of the number of transfusions of blood products in the first 24 h is given in Table 3.

**Table 2:** Type of surgical procedure

Operation	Number (%)	Mean duration of ECC
CABG	373 (74.5 %)	67 ± 26
OPCAB	60 (12 %)	N/A
AVR	50 (10 %)	77 ± 20
MVR/MV repair	17 (3.4 %)	85 ± 35

Data are numbers (%) or mean ± SD

*AVR* aortic valve replacement, *CABG* coronary artery bypass grafting, *ECC* extracorporeal circulation, *MV* mitral valve, *MVR* mitral valve replacement, *OPCAB* off pump coronary artery bypass grafting

**Table 3:** Transfusions of blood products and postoperative serum haemoglobin

RBC transfusions within 24 h	
1–2 units	61 (12.2 %)
3–5 units	14 (2.8 %)
> 5 units	5 (1 %)
Patients with RBC transfusion after 24 h	11 (2.2 %)
Patients with no RBC transfusion during hospital stay	409 (81 %)
Patients with transfusion of fresh frozen plasma	46 (9.2 %)
Patients with transfusion of platelets	23 (4.6 %)
Serum haemoglobin on the 1st postoperative day (mmol/L)	6.4 ± 0.8
Serum haemoglobin on the 3rd postoperative day (mmol/L)	6.8 ± 0.8

Data are presented as numbers (%) or mean ± SD

The number of patients who did not receive any RBCs during hospital stay was 409 (81 %). Sixty-one patients (12.2 %) received 1–2 units of blood and 14 patients (2.8 %) received 3–5 units of blood within the first 24 h after surgery. In all these patients, blood was available within 20 min as planned. Only 11 patients (2.2 %) received RBCs on the ward after 24 h of surgery.

The mean serum haemoglobin was 6.4 ± 0.8 mmol/l (10.3 ± 1.3 g/dL) on the first postoperative day, and 6.8 ± 0.8 mmol/l (11 ± 1.3 g/dL) on the third postoperative day.

Table 3 shows the number of transfused blood products, the level of serum haemoglobin, transfusion of RBCs as well as fresh frozen plasma (FFP) units. The serum haemoglobin levels in the first and third postoperative days are also shown.

Postoperative complications (Table 4) included re-exploration for bleeding (4.6 %), myocardial infarction (3.2 %), cerebrovascular accident (CVA) (0.4 %) and deep sternal wound infection (0.6 %). Results of the univariate logistic regression analysis for the need for blood transfusion are shown in Table 5. Female gender (OR = 3.12) and LVEF <35 % (OR = 2.79) predicted a higher risk of blood transfusion. A higher logistic (OR = 1.06) and a higher additive EuroSCORE (OR = 1.17) are significant predictors for the need of RBC transfusion.

**Table 4:** Postoperative complications

Complication	Incidence
Re-exploration for bleeding	24 (4.6 %)
Myocardial infarction	16 (3.2 %)
CVA	2 (0.4 %)
Deep sternal wound infection	3 (0.6 %)
Operative mortality	1 (0.2 %)

*CVA* cerebrovascular accident

**Table 5:** Univariate logistic regression analysis for predictors of perioperative red blood cell transfusion

Variable	OR (95 % CI)	*p*-value
Age*	1.02 (0.99–1.04)	0.060
Female sex	3.12 (1.86–5.23)	<0.0001
Diabetes	0.89 (0.49–1.61)	0.703
Hypertension	1.44 (0.91–2.30)	0.117
COPD	1.51 (0.73–3.12)	0.258
PVD	0.74 (0.36–1.52)	0.423
LVEF <35 %	2.79 (1.06–7.31)	0.036
Preoperative serum creatinine*	1.00 (0.99–1.00)	0.923
Logistic EuroSCORE*	1.06 (1.00–1.12)	0.043
Additive EuroSCORE*	1.17 (1.06–1.29)	0.001

*OR* odds ratio, *CI* confidence interval, *COPD* chronic obstructive pulmonary disease, *PVD* peripheral vascular disease, *LVEF* left ventricular ejection fraction

* used as a continuous variable

## Discussion

This study demonstrated, in a selected group of patients, that it is safe to perform cardiac surgery without the immediate availability of RBCs in the operating room. This resulted in a considerable reduction in cross-matching and transportation with possible damage of non-used RBC. We used data from an earlier report of our group [4] to identify patients with a relatively low risk for receiving perioperative RBC transfusions.

Transfusion of RBCs is not only associated with an increase in morbidity and mortality, but also with a longer ICU stay and total hospital stay [1]. Moreover, the effect of duration of storage of RBC on morbidity has been addressed [5–10]. In an earlier retrospective analysis of 10,626 patients undergoing cardiac surgery in our institution [2], we found a significant correlation between the number of RBC units received by the patients and the incidence of early mortality. McKenny et al. [9] also found the number of transfused RBC units to be associated with adverse outcome and longer hospital stay after cardiac surgery. Koch et al. [11] found significantly reduced survival among transfused patients compared with non-transfused patients. Both early (6 months) and late hazard phases (up to 10 years) showed that transfusion of red cells is associated with a decreased survival in isolated CABG patients. According to these authors, attention should be directed toward blood conservation methods and a more judicious use of the RBCs [11].

One of the primary rationales of the NERC protocol is to help maintenance of adequate preservation of the RBCs. Non-used units of blood must be returned back to the blood bank for further use. However, the quality of these RBC units is likely to be adversely influenced by the improper reservation in the operation room as well as during transport [12]. The value of transfusion of these RBC units is physiologically less effective and can even increase the incidence of postoperative complications [13–15].

The effect of storage time of RBCs on the outcome after cardiac surgery has gained an increasing interest in recent literature. In an earlier study, storage time of the RBCs was not found to be a significant predictor of early or late mortality after CABG in our centre [5]. The endpoint of that study was all-cause mortality without analysing the effect of storage time of RBCs on morbidity.

On the other hand, Sanders et al. [10] found that patients receiving older blood have an increased incidence of prolonged hospital stay and renal complications compared with those receiving new blood [10]. Koch et al.[6] also found a correlation between transfusion of old blood and mortality and both renal and pulmonary complications after cardiac surgery. The controversy between different reports might be explained by the various patient populations studied, differences in study design or analysis, or different methods of blood storage. [5]

The criteria of selected patients who are candidates of this NERC protocol are of utmost importance. We applied these criteria after studying the risk factors of perioperative transfusion in our centre [4]. These criteria can vary in different centres and according to the availability of RBC units. If the blood is not electively ordered, it must be possible to deliver it on time if urgently needed.

Several blood conservation strategies [16–18] have been proposed in order to improve outcome after cardiac surgery, reduce the need for donor blood and hence lower the overall costs of transfusion. A wide variation in clinical practice and the application of guidelines have been reported in as many as 1402 surveys in 1061 institutions in the United States and Canada [19] Most of these studies examined liberal versus restrictive transfusion practices in cardiac surgery. However, there is no prospective randomised trial comparing the outcomes of a program that practises blood conservation versus the common practice of blood use in cardiac surgery [20]. Our NERC protocol is prospectively designed and the data of patients are prospectively collected as well. Only 19 % of the patients needed blood transfusions during the whole hospital stay. In all these patients, the blood needed was immediately available on site in the hospital. In the present study, the incidence of postoperative complications is comparable with the general incidence of complications in our institution as demonstrated in previous studies of similar patients in our department [2]. Univariate predictors of postoperative need for blood transfusion were found to be gender (female sex), LVEF <35 %, logistic and additive EuroSCORE.

The use of cell saver for shed blood or residual blood is a routine in our daily cardiac surgical practice. Current evidence suggests that the use of a cell saver reduces exposure to allergenic blood products or red blood cell transfusion for patients undergoing cardiac surgery [21].

### Limitations

The results of this observational single-centre study must be interpreted with caution. Our findings were the result of the local protocol agreed upon by a team of anaesthesiologists, ICU physicians, cardiac surgeons and the blood bank. Whether the same results can be applied to other institutions remains to be investigated. The lack of a control group is also a shortcoming. However, matching this group with another group that was operated earlier was not convenient.

## Conclusions

In a selected group of patients, it is safe to perform cardiac surgery without the immediate availability of RBCs in the operating room. Preoperative univariate predictors of the need for postoperative RBC transfusion were female gender, left ventricular function (LVEF) <35 % and the EuroSCORE.
